# Extreme prematurity and perinatal risk factors related to extremely preterm birth are associated with complex patterns of regional brain volume alterations at 10 years of age: a voxel-based morphometry study

**DOI:** 10.3389/fneur.2023.1148781

**Published:** 2023-05-19

**Authors:** Hedvig Kvanta, Jenny Bolk, Lina Broström, Lexuri Fernández de Gamarra-Oca, Nelly Padilla, Ulrika Ådén

**Affiliations:** ^1^Department of Women's and Children's Health, Karolinska Institute, Stockholm, Sweden; ^2^Clinical Epidemiology Division, Department of Medicine, Karolinska Institute, Stockholm, Sweden; ^3^Department of Psychology, Faculty of Health Sciences, University of Deusto, Bilbao, Bizkaia, Spain

**Keywords:** magnetic resonance imaging, extremely preterm, voxel-based morphometry, intraventricular hemorrhage, patent ductus arteriosus, gray matter volume, white matter volume

## Abstract

**Objective:**

Structural brain volumetric differences have been investigated previously in very preterm children. However, children born extremely preterm, at the border of viability, have been studied to a lesser degree. Our group previously analyzed children born extremely preterm at term using voxel-based morphometry. In this study, we aimed to examine regional gray and white matter differences for children born extremely preterm derived from the same cohort during childhood. We also aimed to explore the effect of perinatal risk factors on brain volumes in the same group.

**Methods:**

At 10 years of age, 51 children born extremely preterm (before 27 weeks and 0 days) and 38 term-born controls with high-quality 3.0 Tesla magnetic resonance images were included. Statistical analyses using voxel-based morphometry were conducted on images that were normalized using age-specific templates, modulated, and smoothed. Analyses were also performed in stratified groups of children born extremely preterm in the absence or presence of perinatal risk factors that have previously been shown to be associated with volumetric differences at term.

**Results:**

We found volumetric decreases in gray and white matter in the temporal lobes, gray matter decreases in the precuneus gyri, and white matter decreases in the anterior cingulum for children born extremely preterm (all *p* < 0.001, and *p*_*fwe*_ < 0.05). Gray and white matter increases were predominantly observed in the right posterior cingulum and occipital lobe (all *p* < 0.001, and *p*_*fwe*_ < 0.05). Of the examined perinatal risk factors, intraventricular hemorrhage grades I-II compared with no intraventricular hemorrhage and patent ductus arteriosus ligation compared with no treated patent ductus arteriosus or patent ductus arteriosus treated with ibuprofen led to volumetric differences at 10 years of age (all *p* < 0.001, and *p*_*fwe*_ < 0.05).

**Conclusions:**

Children born extremely preterm exhibit volumetric alterations in a pattern overlapping that previously found at term, where many regions with differences are the main hubs of higher order networks. Some, but not all, risk factors known to be associated with structural alterations at term were associated with alterations at 10 years of age.

## 1. Introduction

In Sweden, 3.3 of 1,000 children are born before 27 weeks and 0 days and thus defined as extremely preterm (EPT) (1). Survival rates are high and have improved during the last decade (1). However, these children still have a high risk for adverse neurological outcomes. At the age of 6.5 years, 33% of children born EPT in Sweden during 2004–2007 had moderate-to-severe cognitive disability (2). Magnetic resonance imaging (MRI) has enabled us to further investigate the brain morphology of children born preterm, but the mechanisms underlying developmental challenges are still not fully understood.

Children born very preterm (before 32 weeks and 0 days) and EPT have reduced brain volumes at a global level during childhood compared with term-born children, and these volumetric reductions have been associated with adverse cognitive outcomes (3, 4). We have previously reported that global gray matter (GM) volume was reduced more than global white matter (WM) volume at term for children born EPT and that the relationship was inverted at 10 years of age (4, 5).

Voxel-based morphometry (VBM) encompasses a voxelwise comparison of brain tissue volumes between patient groups and provides information about where volumetric differences are localized (6). We have previously applied this method at term for children born EPT compared with term-born controls (5). The children born EPT exhibited a pattern of widespread regional volumetric alterations (5). Reduced GM and WM brain volumes were found in the temporal lobes and subcortical structures, whereas enlarged brain volumes were found in regions involved in visual processing (5).

To our knowledge, there are no previous studies investigating only children born EPT with VBM beyond infancy, but very preterm adolescents and young adults have been examined (7–9). Reduced volumes and growth of the temporal lobes are consistent findings for children born very preterm compared with term-born controls both during childhood, adolescence, and adulthood (7, 9). Furthermore, various volumetric alterations within the higher order brain networks are common findings that defy straightforward summary (7, 8). The increased volumes in occipital regions reported in infancy for children born EPT have not been found in assessments of older children born very preterm (8).

It remains to be elucidated whether these patterns of GM and WM alterations found at term are distributed similarly at an older age for children born EPT and whether volumetric differences for children born EPT in childhood are different from those previously observed in children born very preterm.

We have previously examined the effect of perinatal risk factors on brain volumes in children born EPT at term and found that those with intraventricular hemorrhage (IVH) grades I-II, patent ductus arteriosus (PDA) ligation, PDA treated with ibuprofen, and the lowest gestational age (GA) were associated with volumetric differences (5).

Low-grade IVH has been considered to not affect neurodevelopment, but at the time of writing, the body of literature suggests that even low-grade IVH can result in adverse neurodevelopmental outcomes, but to a lesser degree than high-grade IVH (10, 11). Whether PDA ligation affects neurodevelopment or not remains unclear. Studies have indicated adverse neurodevelopmental outcomes for children who underwent surgery for PDA (12), and others show no effect of PDA ligation on neurodevelopment (13). Other groups that have investigated the brain volumes of very preterm children have reported associations between brain volume, PDA ligation, and high-grade IVH (14, 15). There is a lack of studies that focus on the most immature children born EPT in relation to brain volumes and perinatal risk factors beyond infancy.

The main aim of this study was to examine the regional brain volume differences in a cohort of children born EPT compared with term-born controls at 10 years of age using VBM.

The secondary aim was to investigate the effect of perinatal risk factors on regional brain volume at 10 years of age. The risk factors examined were IVH grades I-II, PDA (treated with ibuprofen or ligation), and grade of prematurity.

## 2. Materials and methods

### 2.1. Participants

**Figure 1:**
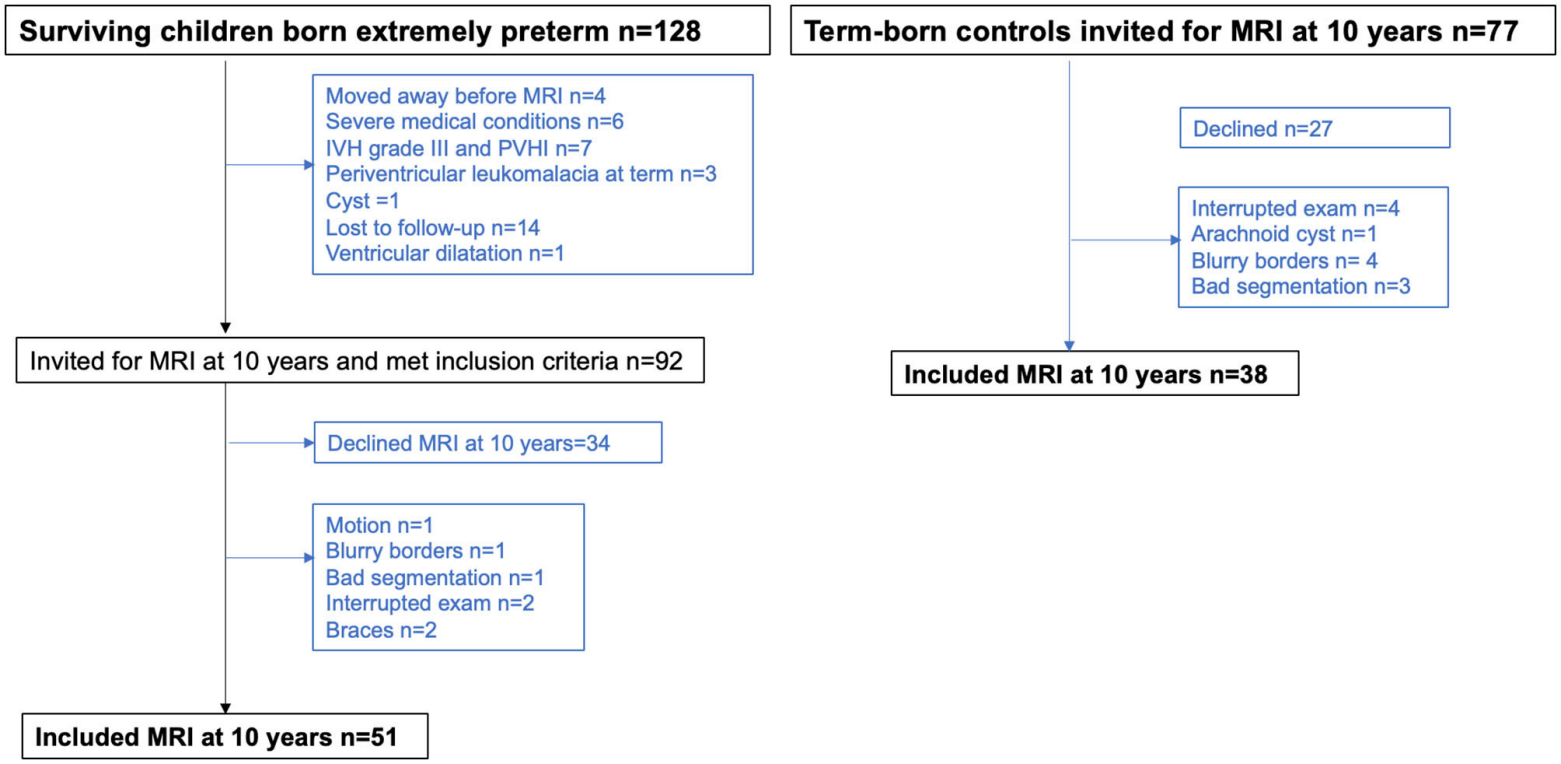
Flow chart of children born extremely preterm and term-born controls. MRI, magnetic resonance imaging.

In this population-based study, 128 children born EPT (defined as birth before GA 27 weeks and 0 days) in Stockholm between 1 January 2004 and 31 March 2007, who survived to term (GA 40 weeks and 0 days) were invited for an MRI examination at ~10 years of age. The cohort is presented in Figure 1 and includes the same children born EPT that were previously described to investigate global brain volumes (4). In total, four children had moved away from the Stockholm area before term MRI, and 14 children were lost to follow-up. Children with cystic periventricular leukomalacia, IVH grade III, periventricular hemorrhagic infarction (previously labeled IVH grade IV), severe white matter abnormalities as defined by a published scoring system (16), focal brain lesions, or severe medical conditions were excluded. In all, six children born EPT were excluded because of severe medical conditions, detailed as follows: one due to Trisomi 21, one had acquired brain lesions, one had hemophagocytic lymphohistiocytosis, and three had congenital malformations (myelomeningocele, esophageal atresia, and vascular malformation). Seven children were excluded due to an interrupted MRI examination, inaccurate segmentation, or low-quality MRI (defined as images with motion artifacts or blurry contrast between GM and WM), leaving 51 children born EPT with high-quality MRI images. In total, nine of the included children born EPT were from multiple pregnancies, where three mothers had two children each included from a multiple pregnancy. Drop-out analyses were performed to compare the 51 children born EPT included with the 41 children excluded due to low quality on MRI or declined participation.

Healthy, singleton, term-born children were randomly selected from the Swedish Medical Birth Registry and matched to children born EPT for place of birth, sex, day of birth, and maternal country of birth at age 2.5 years as part of the National Extremely Preterm Infants in Sweden Study (EXPRESS) (17). The Stockholm-based, full-term children were invited for an MRI examination at ~10 years of age, and of the 77 children who received an invite, 38 had high-quality MRI data and were included in this study. For a summary of the study group, see Figure 1.

This study was approved by the Stockholm Ethics Review Board, and written informed consent was obtained from the parents.

### 2.2. Perinatal risk factors and clinical characteristics

Perinatal information and clinical characteristics were gathered from medical records. Ultrasound scans were performed by a trained neonatologist to detect IVH during the first 3 days after birth, once a week until 27 weeks of gestation, and then every other week until term. Grading was performed according to the Papile classification (18). The definition of PDA in this study was the need for either PDA ligation or treatment with ibuprofen. The treatment decision was at the discretion of the neonatologist in charge (19). In general, children born EPT with a hemodynamically significant PDA according to the preceding echocardiographic evaluation were primarily treated with ibuprofen. The PDA ligation was then indicated if pharmacological therapy failed or when contraindications to ibuprofen were present. The definition of small for gestational age (SGA) was a birthweight < 2 SD below the mean. Bronchopulmonary dysplasia was defined as the need for oxygen at 36 weeks' gestation, and necrotizing enterocolitis was defined using Bell criteria (20).

The effects of perinatal risk factors were investigated by creating subgroups based on the presence or absence of the examined risk factors. The subgroups were as follows: IVH grades I-II or no IVH, PDA ligation or no treated PDA, PDA ligation or PDA treated with ibuprofen, PDA treated with ibuprofen or no treated PDA, and birth **≤**GA 25 + 6 weeks or birth ≥GA 26 + 0 weeks. Risk factors were selected because they have been shown to be associated with altered brain volumes at term in children from the same cohort (5).

### 2.3. MRI acquisition at 10 years of age, preprocessing, and segmentation

Images were acquired using a SIGMA HDx General Electric 3.0-T MRI system (GE Healthcare, Milwaukee, WI, USA), and MRI examinations were performed without sedation. The protocol included a sagittal 3D T1-weighted image with the BRAVO SPGR sequence with a time to inversion of 400 ms, a field of view of 240 × 240 mm^2^, a flip angle of 12°, a slice thickness of 1.0 mm, and a voxel size of 1 × 0.938 × 0.938 mm^3^. All images were assessed by a neuroradiologist.

The T1-weighted images were reoriented in the plane of the anterior commissures, and the skull was stripped using the Brain Extraction Tool from the FMRIB Software Libraries version 5.0.1 (FMRIB Laboratory, University of Oxford, England, UK) (21). Remaining non-brain tissue after automatic brain extraction was removed through manually editing. The criteria to perform manual editing were based on anatomical references and guided by an experienced brain researcher (NP).

The pre-processed images were then automatically segmented into GM, WM, and cerebrospinal fluid (CSF) using the default settings in unified segmentation (22), carried out on the SPM8 software (Wellcome Department, University College, London, UK) using MATLAB version 7.5 (MathWorks, Natick, MA, USA). Age-specific templates from the Template-O-Matic (TOM) toolbox were used (23). The images were spatially non-linearly normalized using DARTEL (24). The segmented images were finally modulated with Jacobian modulation to preserve the amount of volume and subsequently smoothed using a 6 mm Gaussian kernel at full width at half maximum. The aims of smoothing were to improve the signal-to-noise ratio, ensure that the imaging data satisfy a Gaussian distribution, and overcome alignment problems.

Images were inspected before and after each processing step to assess quality. High-quality images were defined as images without motion artifacts, or blurry contrast between GM and WM. Out of the children born EPT with complete MRI data, one child was excluded due to motion artifacts, one had poor contrast between GM and WM, two had braces that generated artifacts, and one image was excluded after unified segmentation since it did not accurately segment brain tissues (Figure 1). Out of the term-born controls with complete MRI data, four children were excluded due to poor contrast between GM and WM and three children were excluded after inspection following unified segmentation due to incorrect classification of brain tissues (Figure 1).

The Easy Volume toolbox was used to calculate the intracranial volume (ICV), total WM, and total GM, and the method has been previously described (4).

### 2.4. Voxel-based morphometry analysis

Segmented, warped, smoothed, and modulated tissues were used for statistical analyses using a two-sample *t*-test design, adjusting for covariates within the VBM toolbox, using the SPM12 software (Wellcome Department, University College, London, UK).

In the first VBM analyses, children born EPT were compared with term-born controls. Both reduced and increased brain volumes for children born EPT were analyzed by inverting the contrast. The analyses were adjusted for sex, age at scan, and ICV. As analyses were adjusted for ICV, they are interpreted as relative volume differences.

Analyses were repeated with only singleton children, and the results are shown in Supplementary material.

Thereafter, the brain volumes of children born EPT with and without perinatal risk factors were compared using VBM. Both reduced and increased brain volumes for children born EPT with the examined perinatal risk factor were analyzed by inverting the contrast. In these analyses, GA, ICV, age at scan, and sex were used as covariates, except when children born EPT **≤**GA 25 + 5 weeks or **≥**GA 26 + 0 weeks were compared where only sex, age at scan, and ICV were adjusted for. When children born EPT treated with PDA ligation were compared with children with no treated PDA or with PDA treated with ibuprofen, we added IVH grades I-II as a covariate. When IVH grades I-II were compared with no IVH, PDA ligation was added as a covariate. However, since these adjustments did not change the results, these covariates were not included in the final analyses.

The initial cluster-forming height threshold was set at *p* < 0.001. All results presented in tables and figures were then family-wise error (FWE) corrected (*p* = 0.05) at cluster level using the Gaussian random field theory to account for multiple comparisons. Absolute threshold masking was set at 0.1 to avoid possible edge effects between tissue types (25). We report the corrected cluster-level *p-*values and their corresponding Montreal Neurological Institute (MNI) peak coordinates. The Harvard-Oxford cortical and subcortical structural atlases (26) and a conversion of the original Talairach Atlas registered to MNI space were used for anatomical orientation (27).

### 2.5. Statistical analysis

All the statistical analyses of clinical characteristics and total brain volumes were performed using SPSS, version 25 (IBM Corp, Armonk, NY, USA). Variables were tested for normality and homogeneity prior to analysis. Normally distributed continuous variables were compared using the two-sample *t*-test, and non-normally distributed variables were compared using the Mann–Whitney *U*-test. Categorical variables were compared using Pearson's chi-squared or Fisher's exact test, as appropriate. The ICV, total GM, and total WM volumes were compared between children born EPT with and without perinatal risk factors. These total brain volumes were first univariately analyzed and then analyzed as dependent variables in a multivariate general linear model with group status (presence or absence of a perinatal risk factor) as independent variables, adjusting for sex, age at scan, and GA. A statistical significance level of *p* < 0.05 was used.

## 3. Results

The clinical characteristics of the children born EPT and term-born controls are summarized in Table 1. Drop-out analyses of the children born EPT included in the study compared with the children declining participation or excluded due to low MRI quality are presented in Supplementary Table 1, and the included children born EPT had a higher GA and a lower rate of bronchopulmonary dysplasia than the children not included.

**Table 1:** Characteristics of the study groups.

	**Children born EPT with MRI at 10 years *n* = 51**	**Term-born controls with MRI at 10 years *n* = 38**	***p*-value**
Gestational age, median (range) weeks	25.6 (23.6–26.6)	40.1 (37.3–41.6)	< 0.001^b^
Birth weight, mean (SD), g	846 (148)	3,739 (454)	< 0.001^a^
Age at MRI, median (range) years	10.3 (9.0–11.8)	10.1 (8.3–11.6)	0.45^b^
Sex male, *n* (%)	24 (47)	19 (50)	0.78^c^
Mothers that attended university, *n* (%)	28/44 (64)	19/30 (63)	0.98^c^
Intracranial volume, mean (SD) cm^3^	1392.6 (113.9)	1449.2 (121.1)	0.027^a^
Head circumference, mean (SD), cm	30/51, 53.3 (2.6)	16/38, 54.3 (1.2)	0.15^a^
Multiple births, *n* (%)	9 (18)	–	–
Patent ductus arteriosus, *n* (%)	36 (71)	–	–
Ibuprofen for PDA, *n* (%)	34 (67)	–	–
Patent ductus arteriosus ligation, *n* (%)	16 (31)	–	–
Small for gestational age, *n* (%)	4 (8)	–	–
Bronchopulmonary dysplasia, *n* (%)	18 (35)	–	–
Any retinopathy of prematurity, *n* (%)	39 (76)	–	–
Intraventricular hemorrhage I–II, *n* (%)	16 (31)	–	–
Necrotizing enterocolitis, *n* (%)	7 (14)	–	–
Antenatal steroids, *n* (%)	48 (94)	–	–

^a^Student's t-test.

^b^Mann–Whitney U.

^c^Pearson's chi-squared.

EPT, extremely preterm; MRI, magnetic resonance imaging.

In Supplementary Tables 2a–e, the clinical characteristics and total brain volumes of the children born EPT with and without perinatal risk factors are presented. Supplementary Table 2a describes the children born EPT with IVH grades I-II, Supplementary Tables 2b–d describes the groups treated for PDA, and Supplementary Table 2e compares children born EPT born **≤**GA 25 weeks and 6 days with those born ≥GA 26 weeks and 0 days. Of the 51 included children born EPT, there were 16 treated with PDA ligation, and 14 out of these were previously treated with ibuprofen. There were 20 children treated with only ibuprofen. Of the analyzed perinatal risk factors, only the children born EPT with PDA ligation had reduced ICV compared with children born EPT with no treated PDA (mean difference 81.8 cm^3^ and 95% confidence interval 2.2–161.4, *p* = 0.044) and children born EPT with PDA treated with ibuprofen (mean difference 80.0 cm^3^ and 95% confidence interval 3.3–156.6, *p* = 0.041), but this did not remain significant after adjusting for sex, age at scan, and GA, see Supplementary Tables 2b, c. No significant differences in ICV, total GM, or total WM brain volumes were found for children born with IVH grades I-II compared with children with no IVH (Supplementary Table 2a), PDA treated with ibuprofen compared with no treated PDA (Supplementary Table 2d), or **≤**GA 25 weeks and 6 days compared with **≥**GA 26 weeks and 0 days (Supplementary Table 2e). IVH grades I-II were more common in children treated with PDA ligation, and PDA ligation was more common in children with IVH grades I-II (Supplementary Tables 2a–c).

### 3.1. Regional volumetric differences for children born EPT compared with term-born controls

**Figure 2:**
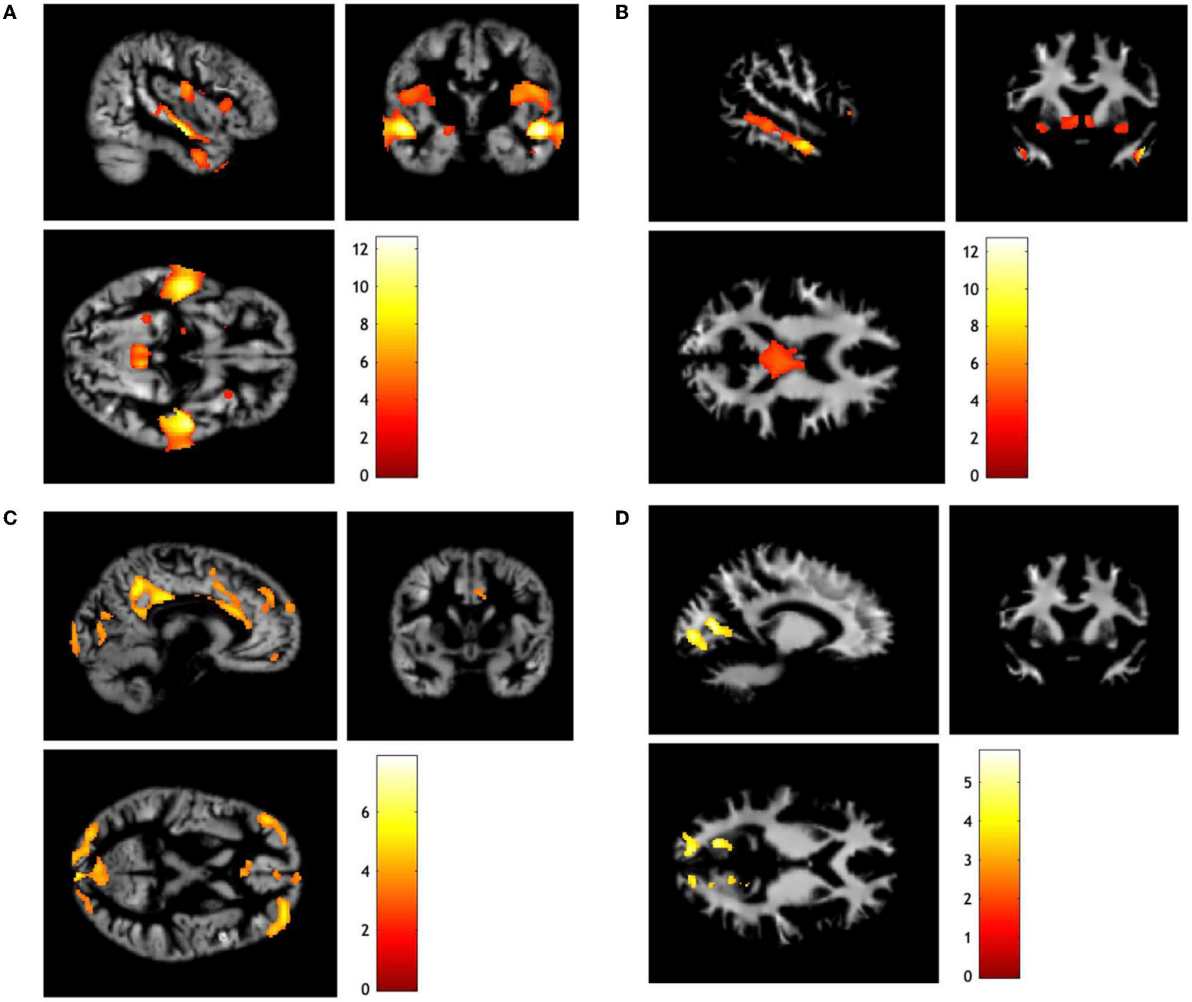
Sagittal, coronal, and axial views of regional volumetric differences for children born extremely preterm compared with term-born controls. **(A)** Gray matter decreased, **(B)** white matter decreased, **(C)** gray matter increased, **(D)** white matter increased. The colored bars represent *t*-score. Differences are mapped on the render of one child born extremely preterm. Analyses are adjusted for sex, age at scan, and intracranial volume. Display orientation right = right. Results are presented with a threshold of *p* < 0.001, with family-wise error correction *p* < 0.05 at the cluster level.

The children born EPT exhibited significant regional volumetric differences with increases and decreases in both GM and WM when adjusted for sex, age at scan, and ICV (Table 2, Figure 2).

**Table 2:** Regional volumetric differences between children born EPT (*n* = 51) and term-born controls (*n* = 38) analyzed with voxel-based morphometry at 10 years of age, adjusted for intracranial volume, sex, and age at scan.

**Anatomical region**	**Hemisphere**	**Cluster, No. voxels**	Cluster level, *P*-value < 0.05^*^	**T statistics**	**Coordinates in MNI152**		
					**x**	**y**	**z**
**EPT**<**term-born**							
**Gray matter**							
Superior temporal gyrus	Right	6,513	< 0.001	12.51	50	−12	−8
Middle temporal gyrus							
Insula							
Superior temporal gyrus	Left	6,966	< 0.001	12.58	−50	−10	−8
Middle temporal gyrus							
Insula							
Temporal fusiform cortex	Right	426	0.041	5.77	41	−6	−29
Precuneus cortex	Right	1,009	< 0.001	7.02	20	−70	31
Precuneus cortex	Left	575	0.012	4.46	−18	−39	42
**White matter**							
Frontal orbital gyrus	Left	491	0.014	5.22	−33	33	−2
Middle temporal gyrus	Right	14,970	< 0.001	12.67	50	−12	−18
Inferior temporal gyrus							
Right thalamus							
Brainstem							
Middle temporal gyrus	Left	1,143	< 0.001	10.56	−48	−9	−18
Anterior cingulum	Right	387	0.034	4.61	12	32	4
Right crus II	Right	452	0.019	5.50	30	−69	−38
**EPT** >**term-born**							
**Gray matter**							
Middle temporal gyrus	Right	1,035	< 0.001	6.77	54	−49	−8
Posterior cingulate gyrus	Right	17,596	< 0.001	7.87	6	−28	34
Cingulate gyrus							
Temporal occipital fusiform gyrus	Right	1,135	< 0.001	7.05	27	−46	−14
Temporal occipital fusiform gyrus	Left	2179	< 0.001	6.98	−31	−48	−12
Occipital pole	Right	2,905	< 0.001	5.87	3	−99	9
Lateral occipital cortex	Left	1,701	< 0.001	5.43	−46	−66	25
Lateral occipital cortex	Right	1,779	< 0.001	5.32	41	−67	27
Lateral occipital cortex	Left	1,129	< 0.001	5.20	−28	−88	9
**White matter**							
Posterior cingulum	Right	1,186	< 0.001	5.81	14	−42	−2
Intracalcarine cortex, occipital lobe	Left	464	0.017	5.80	−15	−72	6
Occipital pole	Left	489	0.014	5.04	−10	−90	3

^*^Threshold of p < 0.001, with family-wise error correction p < 0.05 at cluster level. EPT, extremely preterm; MNI, Montreal Neurological Institute. The regions in the left column refer to the location of the peak coordinates within each cluster and are organized in rostral to caudal order. For larger regions that expand multiple regions, these are listed below.

The most prominent GM reductions were found in the temporal lobes. The large clusters had their peek MNI coordinates in the superior temporal gyri and were distributed bilaterally. The volumetric reduction extended medially to the insula and inferiorly to the middle temporal gyri. However, there were also significant GM reductions in children born EPT in the precuneus cortex bilaterally within the parietal lobe.

The WM reductions for the children born EPT were localized adjacent to the GM reductions and were most prominent in the middle temporal gyri. The large WM cluster with peak coordinates in the right middle temporal gyrus extended into the brainstem and also included the right thalamus, while the left WM cluster was only localized in the temporal lobe. In addition, reduced WM volumes were found in the anterior cingulum and cerebellum.

The most prominent volumetric increases for children born EPT compared with term-born controls for GM were localized in the cingulate gyrus with its peak in the right posterior cingulate cortex. Furthermore, there were GM increases in the lateral occipital lobe with bilateral distribution.

The WM increases were adjacent to the GM increases and involved the cingulum as well as occipital regions. All reported results were significant at *p* < 0.001 and cluster-level *p*_*fwe*_ < 0.05.

Analyses were repeated with only the n=42 singleton children born EPT and added as Supplementary Table 3. As the regions with differences did not change, we chose to keep all children born EPT in the final analyses.

### 3.2. Regional volumetric differences for children born EPT with and without perinatal risk factors

**Figure 3:**
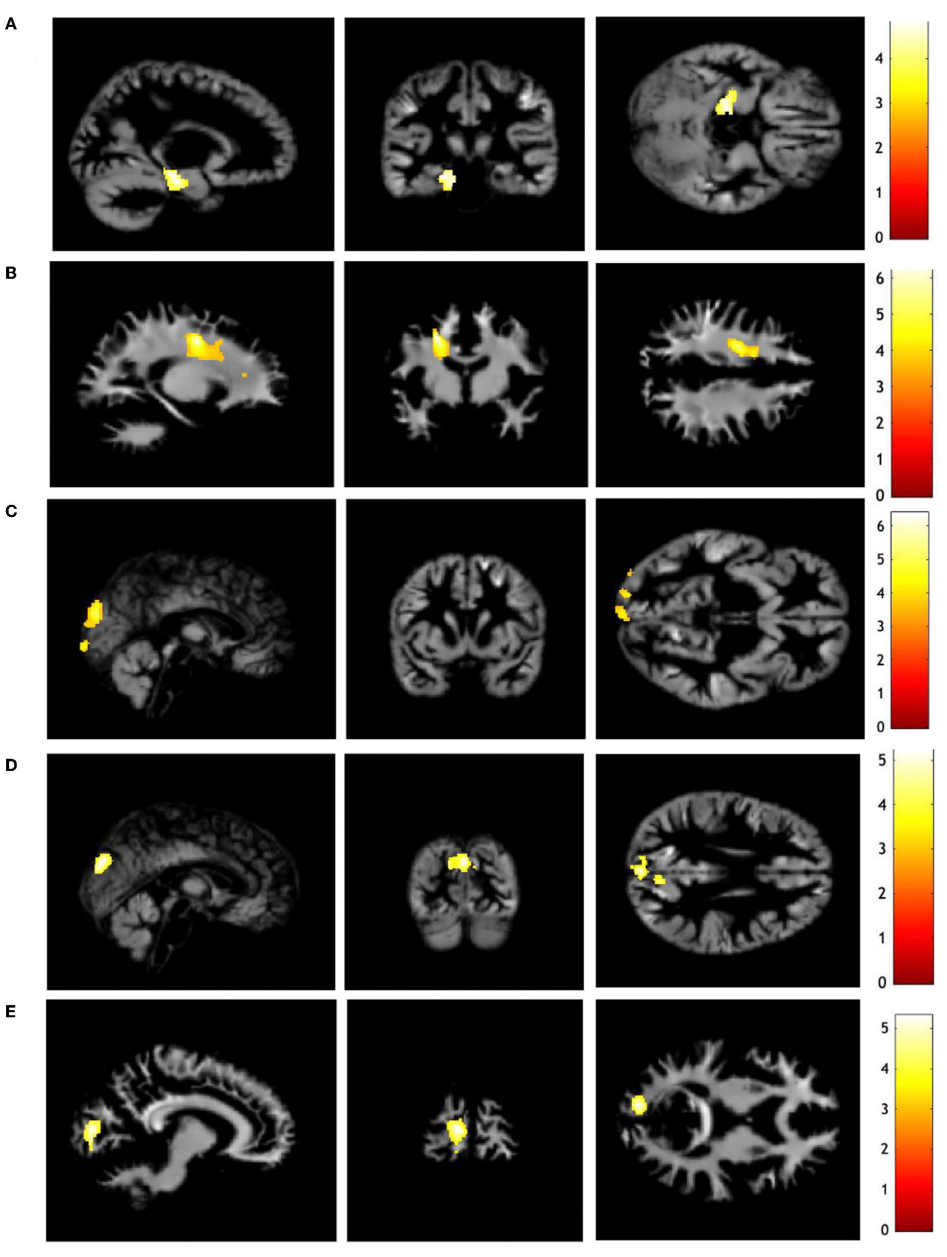
Regional volumetric differences for children born EPT with and without perinatal risk factors. **(A)** Gray matter decreased for IVH grades I-II compared with no IVH, **(B)** white matter decreased for PDA ligation compared with no treated PDA, **(C)** gray matter increased for PDA ligation compared with no treated PDA, **(D)** gray matter increased for PDA ligation compared with PDA treated with ibuprofen, **(E)** white matter increased for PDA ligation compared with PDA treated with ibuprofen. The color bar represents *t*-score. Differences are displayed on the render of one child born EPT. Analyses are adjusted for gestational age, sex, age at scan, and intracranial volume. Display orientation: right = right. EPT, extremely preterm; IVH, intraventricular hemorrhage; PDA, patent ductus arteriosus. Results are presented with a threshold of *p* < 0.001, with family-wise error correction *p* < 0.05 at the cluster level.

All significant regional volumetric differences from analyses using VBM for children born EPT with and without perinatal risk factors are summarized in Table 3 and Figure 3. All reported results were significant at *p* < 0.001, and cluster-level *p*_*fwe*_ < 0.05. Of the investigated perinatal risk factors, PDA ligation and IVH grades I-II resulted in volumetric differences.

**Table 3:** Volumetric differences for children born EPT with and without perinatal risk factors, results were adjusted for sex, gestational age, intracranial volume, and age at scan.

**Neonatal conditions**							
**Anatomical region**	**Hemisphere**	**Cluster, No. voxels**	**Cluster level**, ***P*****-value**<0.05^*^	**T statistics**	**Coordinates in MNI152**		
					**x**	**y**	**z**
**IVH grades I–II** ***n*** = **16**<**no IVH** ***n*** = **35**							
**Gray matter**							
Hippocampus	L	659	0.004	4.85	−15	−24	−15
**PDA ligation** ***n*** = **16**<**no treated PDA** ***n*** = **15**							
**White matter**							
Frontal lobe, premotor area	L	1,394	< 0.001	6.54	−21	9	40
**PDA ligation** ***n*** = **16** > **no treated PDA** ***n*** = **15**							
**Gray matter**							
Lateral occipital cortex	L	1,437	< 0.001	6.38	−36	−81	21
Occipital pole	L&R	1,141	< 0.001	5.06	−3	−99	−3
Occipital pole	L&R	432	0.017	5.28	0	−90	22
Lateral occipital cortex	R	634	0.002	638	47	−63	28
**PDA ligation** ***n*** = **16** > **PDA treated with ibuprofen** ***n*** = **20**							
**Gray matter**							
Occipital lobe	L&R	995	< 0.001	5.23	0	−84	27
**White matter**							
Occipital lobe	L	443	0.012	5.31	−9	−87	9

^*^Threshold of p < 0.001, with family-wise error correction p < 0.05 at cluster level. EPT, extremely preterm; IVH, intraventricular hemorrhage; PDA, patent ductus arteriosus; MNI, Montreal Neurological Institute.

#### 3.2.1. Children born EPT with IVH grades I–II compared with children born EPT with no IVH

Children born EPT with IVH grades I-II had reduced GM volume in the left hippocampus compared with children born EPT without IVH when adjusted for sex, age at scan, GA, and ICV (Table 3 and Figure 3). Children with IVH grades I-II did not have any enlarged regions compared with children without IVH.

#### 3.2.2. Children born EPT treated with PDA ligation compared with children born EPT with no treated PDA

Children treated with PDA ligation had a reduced WM volume in the frontal lobe within the premotor area compared with children with no treated PDA (Table 3 and Figure 3). Multiple enlarged GM regions were observed in the occipital lobe (Table 3 and Figure 3). Adjustments for sex, age at scan, GA, and ICV were made.

#### 3.2.3. Children born EPT treated with PDA ligation compared with children born EPT treated with ibuprofen

GM increases were observed in the occipital lobe when children treated with PDA ligation were compared with children treated with ibuprofen (Table 3 and Figure 3), when adjusted for sex, age at scan, GA, and ICV.

#### 3.2.4. Children born EPT ≤ GA 25 weeks and 6 days compared with ≥GA 26 weeks and 0 days, and children born EPT treated with ibuprofen compared with no treated PDA

Children born EPT ≤ GA 25 weeks and 6 days compared with ≥GA 26 weeks and 0 days did not exhibit any regional volumetric differences after adjustments for sex, age at scan, and ICV. Likewise, treatment with ibuprofen compared with no treated PDA did not result in any regional volumetric differences when adjusted for sex, age at scan, GA, and ICV.

## 4. Discussion

This study demonstrated widespread differences in regional GM and WM volumes for children born EPT compared with term-born controls at 10 years of age. GM decreases were mainly localized bilaterally in the temporal and precuneus gyri, while the GM increases were prominent in the posterior cingulate gyrus on the right side and occipital lobe. The differences in WM and GM followed similar patterns, for example, when the WM decreases were localized in the temporal lobes and the WM increases were found in the occipital lobe and the right posterior cingulate gyrus. Regions with volumetric differences largely overlapped with those previously found at term (5). Of the examined perinatal risk factors known to affect regional brain volumes at term for children born EPT (5), PDA ligation compared with no treated PDA or PDA treated with ibuprofen and IVH grades I-II compared with no IVH resulted in brain volume differences at 10 years of age.

In typically developing children, GM has an “inverted U-shaped” growth pattern (28). The early increase in GM mirrors the rapid synaptogenesis during the third trimester and first years of life, followed by reduced speed of GM growth during childhood, which is associated with synaptic pruning and the myelination at the border between the cortical GM and WM (29–31). The timing of these processes varies for different regions of the brain (29, 30). The WM volume follows a more linear pattern of growth and increases relative to ICV up to the fourth decade of life (32). The increased WM volume during childhood reflects the myelination and maturation of brain connections (28, 29).

One possible explanation for the volumetric differences between children born EPT and term-born controls found in this study could be associated with the early stressor of abrupt extrauterine exposure, potentially modifying the fine-tuned processes occurring in the third trimester, involving premyelinating oligodendrocytes, synaptogenesis, migration, and subplate neurons, thus leading to deviations from typical brain trajectories (31).

Patterns of reduced brain tissue in some regions, accompanied by increased amounts of brain tissue in other regions, are expected because changes in one region often lead to a cascade of alterations in other regions (33).

As expected, WM differences were found adjacent to the GM differences. WM and GM are all components of the same neural circuit and are highly interdependent because there are inseparable connections between neurons, glial cells, and myelin (29).

We found more prominent volumetric increases for GM than for WM volumes when children born EPT were compared with term-born controls. Our group has previously shown that the total GM volume relative to ICV is increased at 10 years of age in the same cohort, while the relative total WM volume is decreased. The findings in this study provide a clearer image of the locations of these volumetric differences (4).

### 4.1. Regional volumetric differences for children born EPT compared with term-born controls

This study suggests selective vulnerability in brain regions, possibly related to the developmental stage in different areas of the brain, as previously suggested (7).

There were bilateral GM volume reductions in the temporal lobes for children born EPT compared with term-born controls. This pattern of volumetric changes matches the alterations previously demonstrated at term (5). Reduced GM volumes in the temporal gyri are supported by previous research investigating older children born very preterm (7, 8, 34), and the temporal lobes have also been shown to have reduced cortical thickness in children born very preterm (35). During the second half of pregnancy, there is a rapid increase in the synaptic density in the temporal lobe, which could be a vulnerable area for exposure to the extrauterine environment (36, 37). There is rapid *in utero* growth of subplate thickness in the superior temporal gyri during the third trimester, and in this study, we observed that this region was particularly affected (38). The temporal gyri are involved in multiple cognitive processes and are thus central to emotional regulation (7). It is understood that children born preterm are at risk for both cognitive and emotional deficits (2, 39). The superior temporal gyri are also critical components of language processing (40–42). Even in the absence of major disabilities, preterm children are more prone to experiencing difficulties in language functions than term-born children (42). Volumetric differences are associated with neurodevelopmental outcomes, such as cognition and language (3).

Interestingly, many of the regions where we found volumetric differences were the main hubs within higher order networks, which are important for sensory integration and socio-emotional regulation, and these hubs are present before birth (43). We know that children born EPT have a high rate of behavioral problems (44, 45). It has been proposed that the main cortical hubs are vulnerable to early adverse events, such as extremely preterm birth (43, 45).

We found reduced GM volume in the precuneus cortex bilaterally in children born EPT and enlarged GM and WM volumes in the posterior cingulate gyrus, which are important hubs within the default mode network (46). We also found reduced WM in the anterior cingulum, an important hub within the salience network, governing the ability to segregate external and internal stimuli and switch the activation between neural networks to guide behavior and attention (47). The observed volumetric alterations in regions within higher order networks are consistent with previous literature on children born very preterm (7, 8). Reduced WM in the anterior cingulum at term was demonstrated previously, indicating a stable alteration throughout childhood (5). We also found WM reductions in the brainstem, which have been observed previously in children derived from this cohort at term and in very preterm children at older assessments (5, 7).

Multiple instances of GM increase were found in the visual areas of the occipital lobe in children born EPT compared with term-born controls, and this was also found at term (5, 48). The enlarged volumes could be due to defect pruning programs or compensatory mechanisms with early onset (33). However, these findings at term may suggest that extrauterine visual exposure in children born EPT has led to increased volumes (5). The underlying mechanisms for these relative volumetric increases and how they relate to visual function are an issue for future investigation. It is important to note that as all the analyses are adjusted for ICV, the volumetric increases reflect the relative size of the specific region in the brain.

Previous research has suggested links between early cerebellar growth and cortical development at older ages in preterm populations (49, 50). Future studies could further investigate the association of early cerebellar growth and the final volumes, with special attention drawn to the regions affected in this study.

### 4.2. Brain volumes for children born EPT with and without perinatal risk factors

Of the examined perinatal risk factors, IVH grades I-II and PDA ligation were associated with brain volume differences.

For children born EPT with IVH grades I-II, the hippocampal volume was reduced on the left side when compared to children born EPT without IVH when analyses were adjusted for sex, GA, age at scan, and ICV. The results remained significant when adjusted for PDA ligation. Recent findings have shown that even low-grade IVH is associated with adverse cognitive outcomes compared with children with no IVH (10, 11). The ICV, total GM, and total WM did not differ for children born EPT with IVH grades I-II when compared to children born with no IVH; thus, the volumetric alterations were limited. Reduced hippocampal volume in less immature children born very preterm with any grade of IVH compared with preterm children without IVH has been previously reported (15). The anatomical proximity of the hippocampus to the ventricles could lead to a particular vulnerability to perinatal events that cause hypoxia and ischemia, such as IVH (51). Reduced hippocampal volumes have been associated with memory performance (15, 51, 52). Reduced hippocampal volume was only significant on the left side of the brain in this study, and preceding research using the same methodology has shown that reduced hippocampal volumes on the left side are associated with memory outcome (52).

At term, IVH grades I-II were associated with both global and regional volumetric differences (5). However, at the regional level, the areas of reduced GM volume were not consistent at term and at 10 years of age, which could indicate adaptational growth and altered brain trajectories (5).

Children born EPT treated with PDA ligation had reduced ICV compared with children with no treated PDA or PDA treated with ibuprofen, but after adjustments for GA, age at scan, and sex, the differences were not significant. When examined with VBM, the children born EPT treated with PDA ligation had reduced WM volume in the premotor cortex within the frontal lobe after adjustments for GA, sex, and ICV, and the results remained significant after adjustments for IVH grades I–II. Surgical closure of the PDA has been associated with neurosensory impairments, including a higher risk of cerebral palsy (12). Importantly, it is not possible to distinguish the risk of the surgery from the risk of hypooxygenation to the brain from a long-lasting hemodynamically significant ductus (14). Enlarged brain volumes were found in the occipital lobe of children treated with PDA ligation compared with children born EPT with no treated PDA or PDA treated with ibuprofen. These volumetric differences could be a sign of reorganization; however, this needs to be elucidated further in future research.

At term age, the children born EPT treated with PDA ligation had reduced brain volumes in multiple brain regions for both GM and WM, including the temporal gyri and cerebellum, when compared to children with no treated PDA and children treated with ibuprofen for PDA, as previously reported (5). Thus, this risk factor was associated with more prominent alterations at term than at 10 years of age, and there may be favorable compensatory growth during childhood.

No volumetric differences were found for children born EPT treated with ibuprofen for PDA compared with children with no treated PDA, while at term, the children born EPT treated with ibuprofen for PDA had enlarged volumes (5).

Interestingly, no global or regional volumetric differences were found for children born **≤**GA 25 weeks and 6 days compared with children born **≥**GA 26 weeks and 0 days. The effect of GA on brain volume is known from the literature (7, 53). However, IVH grades I-II and PDA ligation had larger impacts on regional brain volumes than prematurity *per se* in our homogeneous sample of children born EPT without major brain lesions at 10 years of age.

### 4.3. Strengths and limitations

A strength of this study is its population-based study design and its focus on children specifically born EPT before 27 gestational weeks without major brain lesions. Furthermore, this study extends beyond early childhood, assesses the regional brain volume growth, and evaluates the effect of perinatal risk factors on regional brain volumes.

This study had several limitations. First, because the study population was limited, the stratified groups were small when examining perinatal risk factors. For the same reason, we could not perform separate analyses on children with IVH grade I or IVH grade II only. Likewise, we could not perform sub-analyses of children with unilateral or bilateral IVH or if the IVH was on the right or left side. We could also not stratify children treated with only PDA ligation and children treated with PDA ligation and ibuprofen as the numbers were too few.

Furthermore, PDA treatment protocols and the operating technique for ligation vary between centers and countries, which may limit the generality of the results.

Also, the stratification for GA was made unevenly with children born from 22 weeks and 0 days up to 25 weeks and 6 days in one group, and only children from 26 weeks and 0 days up to 26 weeks and 6 days in the other group. This choice was made as there were too few surviving children with the lowest gestational ages, and to homogenize the stratification with previous analyses done at term.

We are aware that software and pipelines for image processing are constructed for adult brains. We did however use an age-specific atlas to increase the robustness of data averaging. Also, the VBM methodology has previously been used in multiple studies in similar populations (8).

Some of the volumetric differences were large and expanded over more than one brain region, which hampers the anatomical precision. In future, research could further investigate these regions with other methodologies, such as TBSS, or structural covariance.

Another limitation was the inclusion of children born EPT from multiple pregnancies. However, the VBM analyses were repeated with only singleton children and the results remained similar and were added as Supplementary material.

## 5. Conclusion

Children born EPT had altered regional brain volumes at 10 years of age compared with term-born controls. Surgically ligated PDA and IVH grades I-II were associated with alterations in regional brain volume growth. The results emphasize the need to optimize brain growth during the neonatal period and beyond and to limit the exposure to preventable perinatal risk factors. Longitudinal studies, including cognitive performance and language data, would help to determine the significance of these volumetric brain alterations.

## Data availability statement

The raw data supporting the conclusions of this article will be made available by the authors, without undue reservation.

## Ethics statement

The studies involving human participants were reviewed and approved by the Regional Ethical Review Board in Stockholm. Written informed consent to participate in this study was provided by the participants' legal guardian/next of kin.

## Author contributions

HK, JB, NP, LB, and UÅ: conceptualization. HK, LB, LF, NP, and UÅ: methodology. HK, NP, and LF: formal analysis. HK: writing—original draft. HK, JB, LB, LF, NP, and UÅ: writing—review and editing. HK and NP: visualization. UÅ and NP: supervision. UÅ: funding acquisition, data curation, and resources. All authors contributed to the article and approved the submitted version.
