# Supplementary material for: Extreme prematurity and perinatal risk factors related to extremely preterm birth are associated with complex patterns of regional brain volume alterations at 10 years of age: a voxel-based morphometry study
Source: Front Neurol. 2023 May 19;14:1148781. doi: 10.3389/fneur.2023.1148781 (PMC10235462; doi:10.3389/fneur.2023.1148781)
Supplement: Supplementary file 1 [file Table_1.DOCX]

**Supplementary Table 1**

Drop-out analyses for children born EPT not included due to declined participation or low quality on MRI and included children born EPT (n=51+41=92).

|  | **Children born EPT included**  **n=51** | **Children born EPT not included**  **n=41** | ***p-*value** |
| --- | --- | --- | --- |
| Gestational age, median  (range) weeks | 25.6  (23.6-26.6) | 25.1  (23.3-26.6) | ^b^0.024 |
| Birth weight, mean (SD), g | 846 (148) | 791 (172) | ^a^0.11 |
| Sex male, n (%) | 24 (47) | 25 (61) | ^c^0.18 |
| Multiple births, n (%) | 9 (18) | 7 (17) | ^c^0.72 |
| PDA ligation, n (%) | 16 (31) | 14 (34) | ^c^0.78 |
| Ibuprofen for PDA, n (%) | 34 (67) | 30 (73) | ^c^0.50 |
| Intraventricular haemorrhage grade 1-2, n (%) | 16 (31) | 18 (43) | ^c^0.22 |
| Small for gestational age <2SD, n | 4 (8) | 5/36 (12) | ^d^0.51 |
| Any retinopathy of prematurity stage, n (%) | 39 (76) | 32 (78) | ^c^0.28 |
| Necrotizing enterocolitis, n (%) | 7 (14) | 7 (17) | ^c^0.66 |
| Bronchopulmonary dysplasia, n (%) | 18 (35) | 24/40 (60) | ^c^0.023 |
| Antenatal steroids, n (%) | 48 (94) | 38 (93) | ^d^1.0 |

MRI, magnetic resonance imaging; SD, standard deviation, PDA, patent ductus arteriosus; ^a^Student’s t test; ^b^Mann-Whitney U; ^c^Pearson’s chi-squared, ^d^Fisher’s exact test
