# Supplementary material for: Extreme prematurity and perinatal risk factors related to extremely preterm birth are associated with complex patterns of regional brain volume alterations at 10 years of age: a voxel-based morphometry study
Source: Front Neurol. 2023 May 19;14:1148781. doi: 10.3389/fneur.2023.1148781 (PMC10235462; doi:10.3389/fneur.2023.1148781)
Supplement: Supplementary file 2 [file Table_2.DOCX]

| **Children born extremely preterm with high quality MRI at late childhood n=51** | **IVH grades I-II n=16** | **No IVH n=35** | **Mean difference (95% CI)** | **p-value** |
| --- | --- | --- | --- | --- |
| Intracranial volume (SD) cm^3^ | 1365.5 (104.33) | 1405.6 (117.17) | 41.1 (-27.6 – 109.9)  6.0 (-68.6 – 56.6)* | ^a^0.24  ^d^0.85* |
| Grey matter volume, mean (SD) cm^3^ | 734.67 (57.21) | 754.50 (61.24) | 19.8 (-16.6 – 56.2)  4.6 (-38.2 – 29.1)* | ^a^0.28  ^d^0.79* |
| White matter volume, mean (SD) cm^3^ | 437.37 (31.94) | 452.99 (42.93) | 15.6 (-8.6 – 39.8)  1.5 (-21.3 – 24.6)* | ^a^0.20  ^d^0.90* |
| Sex male, n (%) | 5 (31) | 19 (54) |  | ^c^0.13 |
| Mothers that attended university, n (%) | 9/15 (60) | 19/29 (66) | - | ^c^0.72 |
| Gestational age, median (range) weeks | 25.25 (2.6) | 26.10 (3.0) | - | ^b^0.019 |
| Birth weight, mean (SD), g | 814 (151) | 861 (146) | 47 (-43 – 136) | ^a^0.30 |
| Age at scan, mean (SD), years | 10.3 | 10.2 | -0.1(-0.6 – 0.4) | ^a^0.65 |
| Ibuprofen, n (%) | 12 (75) | 22 (63) |  | ^c^0.39 |
| PDA ligation | 11 (69) | 5 (14) | - | ^c^<0.001 |
| Treated PDA | 14 (88) | 22 (62) | - | ^c^0.073 |
| Days on mechanical ventilation, days (range) | 14.5 (0-55) | 3 (0-41) | - | ^b^<0.001 |
| Any ROP | 2 (13) | 10 (29) | - | ^e^0.30 |
| BPD | 9 (56) | 9 (26) | - | ^c^0.021 |
| SGA | 1 (6) | 3 (9) | - | ^e^0.77 |

**Supplementary Table 2a.** **Comparison between children born EPT with IVH grades I-II and no IVH.**

^a^ Student’s t-test, ^b^ Mann-Whitney U, ^c^ Pearsons chi-squared, ^d^ multivariate general linear model, ^e^Fisher’s exact test. *=results adjusted for gestational age, sex, and age at scan. IVH, intraventricular hemorrhage; PDA, patent ductus arteriosus; ROP, retinopathy of prematurity; BPD, bronchopulmonary dysplasia; SGA, small for gestational age.

| **Children born extremely preterm with high quality MRI at late childhood n=51** | **PDA ligation n=16** | **No treated PDA n=15** | **Mean difference (95% CI)** | **p-value** |
| --- | --- | --- | --- | --- |
| Intracranial volume, mean (SD) cm^3^ | 1337.20 (113.38) | 1419.05 (102.5) | 81.8 (2.2 – 161.4)  54.4 (-44.7 – 153.8) * | ^a^0.044  ^d^0.27* |
| Grey matter volume, mean (SD) cm^3^ | 720.61 (64.24) | 761.01 (54.07) | 40.4 (-3.4 – 84.2)  21.5 (-34.2 – 77.2) * | ^a^0.069  ^d^0.43* |
| White matter volume, mean (SD) cm^3^ | 431.25 (36.90) | 457.82 (36.83) | 26.6 (-0.5–53.7)  20.3 (-13.8 – 54.4) * | ^a^0.054  ^d^0.23* |
| Sex male, n (%) | 4 (25) | 6 (40) | - | ^c^0.46 |
| Mothers that attended university, n (%) | 9/14 (64) | 8/13 (62) | - | ^c^0.88 |
| Gestational age, mean (SD) weeks | 24.9 (0.9) | 26.1 (0.5) | 1.1 (0.6 – 1.6) | ^b^<0.001 |
| Birth weight, mean (SD), g | 770 (153) | 850 (142) | 80 (-28 – 19) | ^a^0.14 |
| Age at scan, median (range), years | 10.8 (9.1-11.3) | 10.0 (9.1 – 11.4) | - | ^a^0.81 |
| Days on mechanical ventilation, median (range) | 17 (2-55) | 1 (0-37) | - | ^b^<0.001 |
| Any ROP | 14 (88) | 10 (67) | - | ^c^0.22 |
| IVH grades I-II | 11 | 2 | - | ^e^0.003 |
| BPD, n % | 10 (62) | 4 (27) | - | ^e^0.073 |
| SGA, n % | 1 (6) | 2 (13) | - | ^e^0.60 |

**Supplementary Table 2b. Comparison between children born extremely preterm with PDA ligation and no treated PDA.**

^a^ Student’s t-test, ^b^ Mann-Whitney U, ^c^ Pearsons chi-squared, ^d^ multivariate general linear model, ^e^Fisher’s exact test. *=results adjusted for gestational age, sex and age at scan. PDA, patent ductus arteriosus; ROP, retinopathy of prematurity; IVH,intraventricular hemorrhage; BPD,bronchopulmonary dysplasia; SGA, small for gestational age.

| **Children with high quality MRI at late childhood n=51** | **PDA ligation n=16** | **PDA treated with ibuprofen n=20** | **Mean difference (95% CI)** | **p-value** |
| --- | --- | --- | --- | --- |
| Intracranial volume, meand (SD) cm^3^ | 1337.20 (113.38) | 1417.13 (111.68) | 80.0 (3.3 – 156.6)  -19.3 (-116.5 – 77.9) * | ^a^0.041  ^d^0.69* |
| Grey matter volume, mean (SD) cm^3^ | 720.61 (64.24) | 760.51 (56.31) | 40.3 (-0.6 – 81.1)  -11.2 (-63.6 – 41.3) * | ^a^0.053  ^d^0.67* |
| White matter volume, mean (SD) cm^3^ | 431.25 (36.90) | 454.26 (42.61) | 23.0 (-4.3 – 50.4)  -9.9 (-44.8 – 25.0) * | ^a^0.097  ^d^0.57* |
| Sex male, n (%) | 4 (25) | 14 (70) | - | ^e^0.018 |
| Mothers that attended university, n (%) | 9/14 (64) | 11/17 (65) | - | ^c^0.98 |
| Gestational age, median (range) weeks | 24.8 (23.6 – 26.4) | 26.4 (25.1 – 26.6) | - | ^b^<0.001 |
| Birth weight, mean (SD) | 770 (153) | (904 (126) | 133 (39 – 228) | ^a^0.007 |
| Age at scan, median (range), years | 10.8 (9.1-11.3) | 9.8 (9.0 – 11.8) | - | ^a^0.55 |
| Days on mechanical ventilation, median (range) | 17 (2-55) | 3.5 (0-33) | - | ^b^0.001 |
| Any ROP | 14 (88) | 15 (75) | - | ^c^0.35 |
| IVH grades I-II | 11 (69) | 3 (15) | - | ^e^0.001 |
| BPD, n % | 10 (63) | 4 (20) | - | ^e^0.018 |
| SGA, n % | 1 (6) | 1 (5) | - | ^e^1.0 |

**Supplementary Table 2c. Comparison between children born extremely preterm with PDA ligation compared to PDA treated with ibuprofen.**

^a^ Student’s t-test, ^b^ Mann-Whitney U, ^c^ Pearsons chi-squared, ^d^ multivariate general linear model, ^e^Fisher’s exact test. *=results adjusted for gestational age, sex and age at scan. PDA=patent ductus arteriosus, ROP=retinopathy of prematurity, IVH=intraventricular hemorrhage, BPD=bronchopulmonary dysplasia, SGA=small for gestational age.

| **Children born extremely preterm with high quality MRI at late childhood n=51** | **PDA treated with ibuprofen n=20** | **No treated PDA n=15** | **Mean difference (95% CI)** | **p-value** |
| --- | --- | --- | --- | --- |
| Intracranial volume, mean (SD) cm^3^ | 1417.13 (111.68) | 1419.05 (102.53) | 1.9 (-76.8 – 73.1)  53.0.4 (-114.0 – 7.9)* | ^a^0.96  ^d^0.085* |
| Grey matter volume, mean (SD) cm^3^ | 760.87 (56.31) | 761.01 (54.07) | 0.14 (-38.5 – 38.2)  26.1 (-57.1 – 4.9) * | ^a^0.99  ^d^0.096* |
| White matter volume, mean (SD) cm^3^ | 454.26 (42.61) | 457.82 (36.83) | 3.6 (-31.5 – 24.4)  21.5 (-44.9 – 2.0) * | ^a^0.79  ^d^0.071* |
| Sex male, n (%) | 14 (70) | 6 (40) | - | ^c^0.076 |
| Mothers that attended university, n (%) | 11/17 (65) | 8/13 (62) | - | ^c^0.86 |
| Gestational age, median (range) weeks | 26.4 (25.1 – 26.6) | 26.2 (25.2 – 26.6) | - | ^b^0.80 |
| Birth weight, mean (SD) | 904 (126) | 850 (147) | 53 (-39 – 146) | ^a^0.25 |
| Age at scan, median (range), years | 10.2 (0.9) | 10.2 (0.8) | 0.07 (-0.6- 0.5) | ^a^0.80 |
| Days on mechanical ventilation, median (range) | 3.5 (0-33) | 1 (0-37) | - | ^b^0.052 |
| Any ROP | 15 (75) | 10 | - | ^c^0.59 |
| IVH grades I-II | 3 (15) | 2 (13) | - | ^e^1.0 |
| BPD, n % | 4 (20) | 4 (27) | - | ^e^1.0 |
| SGA, n % | 1 (5) | 2 (13) | - | ^e^0.56 |

**Supplementary Table 2d. Comparison between children born extremely preterm treated with ibuprofen for PDA compared to no treated PDA.**

^a^ Student’s t-test, ^b^ Mann-Whitney U, ^c^ Pearsons chi-squared, ^d^ multivariate general linear model, ^e^Fisher’s exact test. *=results adjusted for gestational age, sex and age at scan. PDA, patent ductus arteriosus; ROP, retinopathy of prematurity; IVH, intraventricular hemorrhage; BPD, bronchopulmonary dysplasia; SGA, small for gestational age; ROP, retinopathy of prematurity.

| **Children born extremely preterm with high quality MRI at late childhood n=51** | **Gestational age ≤ 25+6 n=26** | **Gestational age ≥ 26+0 n=25** | **Mean difference (95% CI)** | **p-value** |
| --- | --- | --- | --- | --- |
| Intracranial volume, mean (SD) cm^3^ | 1385.12 (123.74) | 1400.46 (104.66) | 15.3 (-80.0 – 49.3)  30.8 (-84.8 – 23.3)* | ^a^0.64  ^d^0.26 |
| Grey matter volume, mean (SD) cm^3^ | 745.42 (66.81) | 751.25 (53.59) | 5.8 (-40.0 – 28.3)  13.6 ( -42.7 – 15.5)* | ^a^0.73  ^d^0.35 |
| White matter volume, mean (SD) cm^3^ | 445.75 (42.44) | 450.52 (38.36) | 4.7 (-27.6 – 18.0)  -9.7 (-29.1 – 9.8)* | ^a^0.68  ^d^0.32 |
| Sex male, n (%) | 14 (54) | 10 (40) | - | ^c^0.32 |
| Mothers that attended university, n (%) | 13/23 | 15/21 | - | ^c^0.31 |
| Gestational age, median (range) weeks | 25.3 (23.6 – 25.6) | 26.4 (26.1 – 26.6) | - | ^b^<0.001 |
| Birth weight, mean (SD) | 782 (133) | 912 (134) | 131 (-206 - -56) | ^a^<0.001 |
| Age at scan, median (range), years | 10.3 (0.8) | 10.2 (0.9) | 0.08 (-0.4- 0.5) | ^b^0.74 |
| Days on mechanical ventilation, median (range) | 13.5 (0-55) | 2 (0-19) | - | ^b^0.001 |
| Any ROP | 23 (88) | 16 (64) | - | ^c^0.04 |
| PDA ligation | 13 (50) | 3 (12) | - | ^e^0.006 |
| IVH grades I-II | 12 (46) | 3 (12) | - | ^e^0.03 |
| BPD, n % | 13 (50) | 5 (20) | - | ^c^0.03 |
| SGA, n % | 2 (8) | 2 (8) | - | ^e^1.0 |

**Supplementary Table 2e. Comparison between children born ≤ 25 weeks and 6 days and children born ≥ 26weeks and 0 days.**

^a^ Student’s t-test, ^b^ Mann-Whitney U, ^c^ Pearsons chi-squared, ^d^ multivariate general linear model, ^e^Fisher’s exact test. *=results adjusted sex and age at scan. ROP, retinopathy of prematurity; PDA, patent ductus arteriosus; IVH=intraventricular hemorrhage; BPD, bronchopulmonary dysplasia; SGA, small for gestational age.
