# Supplementary material for: Extreme prematurity and perinatal risk factors related to extremely preterm birth are associated with complex patterns of regional brain volume alterations at 10 years of age: a voxel-based morphometry study
Source: Front Neurol. 2023 May 19;14:1148781. doi: 10.3389/fneur.2023.1148781 (PMC10235462; doi:10.3389/fneur.2023.1148781)
Supplement: Supplementary file 3 [file Table_3.DOCX]

| Anatomical region | hemisphere | Cluster  No. voxels | Cluster level  P value <0.05* | T statistics | Coordinates in MNI152 | | |
| --- | --- | --- | --- | --- | --- | --- | --- |
|  |  |  |  |  | x | y | z |
| **EPT<term-born** |  |  |  |  |  |  |  |
| **Gray matter** |  |  |  |  |  |  |  |
| Superior temporal gyrus  Middle temporal gyrus  Insula | Right | 5404 | <0.001 | 12.13 | 50 | -11 | -8 |
| Superior temporal gyrus  Middle temporal gyrus  Insula | Left | 5586 | <0.001 | 11.53 | -48 | -10 | -8 |
| Precuneus cortex | Right | 735 | 0.003 | 6.42 | 20 | -70 | 31 |
| Cerebellum, anterior lobe | Right | 1902 | <0.001 | 1902 | 6 | -46 | -5 |
| **White matter** |  |  |  |  |  |  |  |
| Middle temporal gyrus  Inferior temporal gyrus  Right thalamus  Brainstem | Right | 10553 | <0.001 | 12.78 | 50 | -10 | -18 |
| Middle temporal gyrus | Left | 828 | 0.001 | 9.67 | -48 | -9 | -18 |
| Anterior cingulum | Right | 591 | 0.005 | 5.42 | 21 | -73 | -35 |
| **EPT >term-born** |  |  |  |  |  |  |  |
| **Gray matter** |  |  |  |  |  |  |  |
| Middle temporal gyrus | Right | 862 | 0.001 | 5.82 | 54 | -49 | -8 |
| Posterior cingulate gyrus  Cingulate gyrus | Right | 4169 | <0.001 | 7.88 | 8 | -27 | 33 |
| Posterior cingulate gyrus  Cingulate gyrus | Left | 6056 | <0.001 | 6.50 | 9 | 32 | 36 |
| Temporal occipital fusiform gyrus | Right | 709 | 0.004 | 6.23 | 27 | -45 | -14 |
| Temporal occipital fusiform gyrus | Left | 1250 | <0.001 | 6.66 | -31 | -48 | -12 |
|  |  |  |  |  |  |  |  |
| Occipital lobe | Right | 3228 | <0.001 | 5.87 | 3 | -99 | 6 |
| Lateral occipital cortex | Right | 1199 | <0.001 | 5.52 | -31 | -76 | 51 |
| Lateral occipital cortex | Left | 990 | 0.001 | 4.89 | -28 | -87 | 9 |
| **White matter** |  |  |  |  |  |  |  |
| Occipital lobe | Left | 679 | 0.003 | 5.75 | -12 | -76 | 10 |
| Occipital lobe | Right | 1348 | <0.001 | 5.55 | 9 | -70 | 13 |
| Occipital lobe | Left | 600 | 0.005 | 5.48 | -13 | -88 | 1 |

**Supplementary Table 3. Regional volumetric differences between singleton children born EPT (n=42) and term-born controls (n=38)** **analyzed with voxel-based morphometry at 10 years of age, adjusted for intracranial volume, sex and age at scan.**

*Threshold of *p*<0.001, with family-wise error correction *p*<0.05 at cluster level. EPT, extremely preterm; MNI, Montreal Neurological Institute. The regions in the left column refer to the location of the peak coordinates within each cluster and are organized in rostral to caudal order. For larger regions that expand multiple regions these are listed below.
